# The combined effects of teachers’ caring behavior and academic self-efficacy on the academic achievement of Chinese undergraduates: a fsQCA approach

**DOI:** 10.1186/s40359-026-05227-5

**Published:** 2026-07-24

**Authors:** Chaoyong Tang, Yongzhi Shi, Fan Jiang, Wanming Chen

**Affiliations:** 1 School of economics and management, Hebei Agricultural University, No. 2596, lekainan street, Lianchi District, Baoding City, Hebei Province 071001 China; 2 Modern Educational Technology Center, Hebei Agricultural University, Baoding City, Hebei Province 071001 China; 3 School of Economics and Management, Nanjing University of Aeronautics and Astronautics, Nanjing City, Jiangsu Province 210016 China

**Keywords:** Teachers' caring behavior, Academic self-efficacy, Academic achievements, Combination configuration, fsQCA

## Abstract

Existing literature focuses on the influence of single or multiple factors on college students’ academic achievement, and few studies analyze the synergistic effect of multiple factors on college students’ academic achievement. Taking 276 college students as samples, this study used the fuzzy set qualitative comparative analysis method to explore the linkage effect of 5 antecedents on college students’ academic achievement from the two aspects of teachers’ caring behavior and academic efficacy. The results show that conscientiousness, supportiveness, inclusiveness, academic ability efficacy and academic behavior efficacy are not necessary conditions for the high or nonhigh academic achievement, and single anthems have weak explanatory power for the academic achievement. There are three ways to drive college students’ academic achievement, namely, the supportiveness-ability efficacy driving model, the conscientiousness-supportiveness-behavior efficacy driving model, and the conscientiousness-efficacy driving model. There are two paths leading to nonhigh academic achievement, and they are asymmetrical with the path of academic achievement improvement. By unpacking the configurational causal relationships among teachers’ caring behavior, academic self-efficacy, and academic achievement, this study enriches extant literature through the fsQCA approach. It further delivers theoretical underpinnings and actionable suggestions for improving college students’ academic performance.

## Introduction

Compared with the basic education stage, the university phase entails substantial changes in curriculum design, learning approaches, motivational orientations, and interpersonal relationships. This period not only represents a critical stage for the development of individuals’ academic competencies but also constitutes a vital window for talent cultivation. As a key theme in educational psychology and an essential component in the assessment of talent development outcomes, academic achievement has long attracted extensive attention from both psychological and educational research communities [1]. Academic achievement is conceptualized as the capacity not only to complete academic requirements but also to possess the competencies necessary for future work, learning, and life [2]. Existing studies have indicated that academic achievement serves not only as an important criterion for evaluating the quality of higher education development but also as a critical indicator for assessing students’ holistic development across the five domains of education [3]. Furthermore, academic performance has been found to significantly enhance university students’ employment remuneration [4] and employment quality [5]. Therefore, promoting academic achievement carries substantial practical significance. A vital prerequisite lies in formulating empirically informed strategies to clarify the configurational causal mechanisms behind academic achievement.

According to social cognitive theory (SCT), academic achievement results from the interplay of internal and external factors [6, 7]. On the one hand, as an external determinant, teacher behavior has been shown to significantly predict academic achievement—for instance, through teacher support [8, 9], teacher-student relationships [10], and teacher collaboration [11]. As an emerging line of inquiry within teacher behavior research, teacher caring behavior is conceptualized as a comprehensive set of actions through which teachers, in their efforts to foster constructive teacher-student relationships, conscientiously fulfill instructional responsibilities, devote time to supporting student development, and demonstrate tolerance toward students [12]. Empirical studies employing hierarchical regression analysis and structural equation modeling have provided evidence that teacher caring behavior exerts a strong explanatory effect on the academic achievement of primary and secondary school students [13–17]. On the other hand, as an internal factor, self-efficacy refers to learners’ subjective judgment and perceived capability regarding their ability, confidence, and strategies for accomplishing learning tasks, encompassing both academic ability self-efficacy and academic behavioral self-efficacy [18]. A substantial body of research indicates that self-efficacy positively and significantly predicts academic achievement [19–22]. However, a literature review reveals inconsistent, and in some cases conflicting, findings regarding the relationship between teacher caring behavior (or teacher support)/academic self-efficacy and academic achievement. For instance, Zhou and Kang [23] found that the direct effects of various dimensions of teacher support on English academic performance were statistically non-significant. Similarly, inconsistent results have been reported for the relationship between academic self-efficacy and academic achievement. Sætre and Sigmundsson [24], in a study of Norwegian upper secondary schools, found that self-efficacy significantly predicted academic achievement only in physical education, but not in general learning or vocational learning contexts. Chen [25] arrived at comparable conclusions. Moreover, prior studies have predominantly focused on the net effects of individual internal or external factors—such as teacher caring behavior and self-efficacy [13–17, 19–22]—on academic achievement, while largely neglecting the combined effects of multiple factors. In addition, most existing research has examined only unidirectional relationships between specific factors and academic achievement, with limited attention to reciprocal relationships. It should also be noted that the majority of these studies have been situated in the basic education context, leaving a notable paucity of empirical evidence specific to higher education. Collectively, these limitations point to significant gaps in the literature that warrant further investigation.

Driven by the limitations and literature gaps identified above, the present study adopted a configurational approach—specifically, fuzzy-set qualitative comparative analysis (fsQCA)—to explore the combined effects of the focal factors on academic achievement. fsQCA derives configurations from empirical case data [26]. By simultaneously considering multiple interdependent factors, this approach overcomes the limitations of net-effect thinking inherent in traditional methods, accommodates causal complexity [27, 28], and yields more compelling explanations [29]. fsQCA is particularly well-suited for investigating the configurational conditions underlying a given outcome [30], while circumventing the constraints of regression-based methodologies noted above [31]. Notably, the application of configurational approaches such as fsQCA in the field of education has gained initial traction. Sheng et al. [32] employed fsQCA to derive five pathways to improved academic performance from the factors of academic burnout, academic buoyancy, and enjoyment. Chun et al. [33], using a combination of PLS-SEM and fsQCA, uncovered nonlinear relationships among college students’ AI literacy, creativity, self-efficacy, and academic achievement. Mustapha et al. [34] utilized fsQCA to identify multiple combinatorial pathways leading to superior academic performance. Accordingly, this method is well-suited to address the aforementioned gaps—namely, the inconsistent findings regarding the relationships between the focal factors and academic achievement, and the neglect of their combined effects on academic outcomes.

This study contributes to the existing literature in three respects. First, it provides empirical evidence on the relevance of teacher caring behavior and academic self-efficacy to academic achievement from a higher education perspective. Second, it examines the joint effects of these two factors on academic achievement. Third, from a methodological standpoint, it employs fsQCA. Finally, this study proposes differentiated contextual perspectives and targeted strategies for fostering high academic achievement among university students.

## Theoretical analysis and research framework

### Social cognition theory

SCT posits that human behavior, individual cognition, and the environment interact bidirectionally, forming a triadic reciprocal determinism model [6, 35]. Within this framework, the environment serves as an external condition that influences behavior, while individual cognition functions as an internal condition that also shapes behavior. Moreover, the environment affects individual cognition as an external antecedent, and in turn, individual cognition, driven by personal needs, interprets and modifies the environment [6, 35].

According to SCT, personal cognitive factors and environmental factors jointly contribute to individuals’ agentic thought, behavior, and emotion [6], which in turn significantly influence their capacity to attain desired outcomes [6, 35]. A primary cognitive factor is self-efficacy [6]. Meanwhile, in the academic context, teachers’ caring behavior constitutes a key environmental element [13]. Self-efficacy refers to an individual’s belief in their capability to accomplish a given task [6, 33]. In academic settings, self-efficacy substantially affects students’ choice of tasks—including those central to their academic achievement—and the self-regulatory processes required to execute them [36]. Moreover, students with higher self-efficacy tend to exhibit greater confidence and cognitive readiness, and are more proactively engaged in the learning process, thereby enhancing their academic performance [37]. Extensive research has further demonstrated that academic self-efficacy positively influences students’ goal-setting, effort expenditure, and persistence [38], which in turn shapes learning motivation, learning behaviors, and academic achievement [39], and serves as a critical condition for attaining favorable academic outcomes [21, 22]. Other work has also shown self-efficacy to be highly correlated with, and uniquely predictive of, achievement [40]. Similarly, students with a heightened sense of self-efficacy are more likely to embrace challenging tasks [41].

With regard to environmental factors that support human agency, students’ sense of relatedness with others emerges when they care for and accept others, and perceive being cared for and accepted in return [42]. Teacher caring behavior is conceptualized as a buffer against stress and risk, a crucial source of academic assistance, an emotional anchor in daily life, an energizing function, and a foundation for socio-emotional development and self-fulfillment [43, 44], each of which elicits positive behavioral and emotional responses [12, 13, 45, 46]. Research has shown that students who experience a strong overall sense of relatedness demonstrate heightened behavioral and emotional engagement at school [47] and achieve higher academic outcomes [17]. When examining student–teacher relationships in the instructional context, evidence indicates that relational support from teachers accounts for substantial variance in educational outcomes [8, 9]. In a case study, Lee and Yuen [48] found that teacher caring positively predicted achievement outcomes.

### Teachers’ caring behavior and academic achievement

Teachers’ caring behavior encompasses educators’ conscientious fulfillment of instructional responsibilities, dedicated investment in student development, and empathetic understanding of learners’ needs, all aimed at fostering positive teacher-student relationships and achieving educational objectives [12, 13]. This multidimensional construct comprises three key elements: conscientiousness—rigorous adherence to curricular standards and high-quality task implementation; supportiveness—strategic allocation of time, knowledge, and resources to cultivate students’ moral, intellectual, physical, aesthetic, and vocational growth; and inclusiveness—holistic acceptance of students’ emotional, cognitive, and behavioral characteristics. Conscientiousness centers on a sense of responsibility, embodying teachers’ fulfillment of fundamental instructional duties and the construction of reciprocal rights and obligations between teachers and students. Supportiveness emphasizes resource provision—including knowledge delivery, time devotion, and competence investment—to facilitate students’ growth, whereas inclusiveness prioritizes psychological acceptance, manifested as respect for individual dignity, accommodation of academic disparities, and recognition of diverse learning styles. Though all three dimensions fall under teacher caring behavior, they bear distinct theoretical demarcations in functional scope. Academic achievement, defined as the comprehensive integration of learning behaviors, attitudes, and outcomes within specific educational contexts [20], serves as a critical metric for evaluating educational effectiveness. It extends beyond knowledge acquisition to encompass value cultivation, skill development, and innovative capacity enhancement. Notably, such achievement not only reflects students’ academic competence but also significantly influences their career trajectories. As a core indicator of educational quality, it provides essential feedback for optimizing pedagogical practices and institutional training programs.

Empirical studies demonstrate that teachers’ professional conduct—manifested through instructional approaches, work ethic, pedagogical encouragement, and student respect—serves dual functions in educational contexts: fostering constructive teacher-student interactions and enhancing learning outcomes [12]. These behavioral dimensions optimize educational influence by unlocking learning potential, stimulating proactive dispositions, and improving academic performance, establishing teacher care as a critical determinant of collegiate success.

The Self-Determination Theory (SDT) elucidates the mechanism underlying teachers’ caring behavior and academic outcomes [1, 8, 13]. The SDT [49] posits that individuals have three basic psychological needs—autonomy (the sense that one’s actions are volitional), competence (the ability to effectively interact with one’s environment), and relatedness (the establishment of meaningful connections with others). When the social environment (e.g., teacher behavior) satisfies these three needs, individuals’ motivation is activated or effectively internalized, thereby promoting high-quality engagement, persistence, and psychological well-being [50]. Conversely, when these needs are thwarted, motivation declines and maladaptive functioning may ensue. According to the SDT, teacher caring behavior influences academic self-efficacy and academic achievement through the differentiated satisfaction of students’ basic psychological needs. Specifically, (1) Conscientiousness and academic achievement. Teacher conscientiousness—manifested in clear structure and timely feedback—primarily satisfies the need for competence, thereby directly enhancing students’ confidence in mastering knowledge (academic ability self-efficacy). Teachers’ dutiful implementation of pedagogical responsibilities ensures students’ access to three critical resources: (1) Curricular expertise, (2) Diversified instructional methods, and (3) Developmental opportunities—all significant predictors of academic achievement [13]. Through perceiving educators’ professional dedication and emotional investment, students internalize learning motivations, transforming external expectations into self-regulated scholarly pursuits. (2) Supportiveness and academic achievement. Teacher supportiveness—manifested through offering choices and respecting autonomy—directly satisfies students’ need for autonomy, leading them to attribute academic success to their own efforts and strategies rather than to external coercion, thereby significantly enhancing academic self-efficacy. High self-efficacy, in turn, drives students to adopt more self-monitoring and persistence strategies, increases learning engagement [50], and ultimately contributes positively to academic achievement. (3) Inclusiveness and academic achievement. Teacher inclusiveness—expressed through error acceptance and a safe classroom climate—primarily satisfies the need for relatedness, which indirectly bolsters both types of efficacy by reducing anxiety and promoting help-seeking behaviors. Perceived respect and belongingness catalyze students’ voluntary participation in academic activities. Teachers’ empathetic understanding establishes reciprocal teacher-student relationships while strengthening emotional investment and collective identity [51]. This relational dynamic enhances cognitive absorption in learning tasks and improves performance metrics through heightened institutional commitment.

The tripartite model of teachers’ caring behavior—supportiveness, inclusivity, and conscientiousness—operates through interconnected psychological and practical mechanisms. By fulfilling students’ basic psychological needs and providing strategic academic scaffolding, these behaviors not only enhance immediate learning outcomes but also cultivate sustainable competencies in value internalization, skill acquisition, and innovative thinking. This evidence positions teacher care as both an emotional enabler and pedagogical imperative in higher education contexts.

### Academic self-efficacy and academic achievement

Academic self-efficacy denotes learners’ self-assessment of their capacity to achieve educational objectives, encompassing two dimensions: *academic capability self-efficacy* (confidence in completing tasks and avoiding failure) and *academic behavioral self-efficacy* (assurance in implementing effective learning strategies) [6, 20]. Empirical evidence indicates a positive correlation between self-efficacy levels and academic goal attainment, with higher efficacy fostering enhanced motivation, persistence, and performance optimization [13, 52].

Self-efficacy theory posits that individuals’ ability evaluations directly influence behavioral choices [53]. Students with robust academic capability self-efficacy exhibit heightened intrinsic motivation, proactive learning dispositions, and cognitive engagement [54]. This self-belief system translates into increased time investment and strategic resource allocation during learning processes, thereby improving achievement outcomes [51]. The behavioral dimension of self-efficacy demonstrates dual functionality: (1) Cognitive Activation: It drives students to critically evaluate and refine learning approaches when confronting challenges [52]; (2) Competency Reinforcement: Through cross-disciplinary self-assessment, learners develop adaptive problem-solving skills and academic resilience [36]. Notably, both dimensions synergistically contribute to academic success through distinct mechanisms. Capability efficacy strengthens foundational confidence in subject mastery, while behavioral efficacy enhances metacognitive regulation and methodological flexibility. This dual pathway explains why self-efficacious learners demonstrate superior performance in complex educational environments, particularly in tasks requiring creative thinking and iterative improvement.

### Model construction

Existing research on the effects of internal and external factors—such as teacher caring behavior and academic self-efficacy—on academic achievement provides a rationale for the selection of conditions in this study. The documented “net effects” of these multifaceted factors on academic achievement also lay a foundation for investigating pathways to its improvement. However, prior studies have not elucidated the interactive relationships among internal and external factors, nor their configurational effects on academic achievement. In reality, the drivers of academic achievement span multiple levels, and these factors interact synergistically to shape academic outcomes.

According to social cognitive theory, academic achievement is shaped by both internal factors and external constraints [8, 9, 13], and is not determined by any single factor in isolation [6]. That is, neither teacher caring behavior alone [48] nor academic self-efficacy alone [53] sufficiently explains students’ academic achievement. Therefore, a systematic investigation into the pathways to academic achievement is warranted. Among these cross-level factors, those at different levels interact with one another. When college students possess a high sense of academic ability efficacy, they tend to maintain positive learning motivation and confidence to persist in improving their academic performance [50], even when current outcomes fall short of expectations. Thus, when students perceive teacher caring behavior—for instance, sensing teachers’ responsibility and commitment, understanding the integration of teaching and learning, and recognizing their own learning tasks—coupled with the intrinsic drive generated by high academic ability efficacy, academic achievement is enhanced through the synergistic effects of internal and external factors. Conversely, when academic self-efficacy is low, even though teacher caring behavior—through its conscientious, supportive, and inclusive qualities—may stimulate learning enthusiasm, increase learning engagement, and reduce learned helplessness, thereby contributing to academic improvement, students may still lack autonomous learning due to insufficient intrinsic motivation, ultimately hindering the enhancement of academic achievement [13]. Furthermore, teacher caring behavior can create a favorable learning context for college students and may alter individual affective states (e.g., learning efficacy), thereby changing behavioral outcomes (i.e., academic achievement) [55]. In addition, academic self-efficacy may in turn influence teacher caring behavior. From the perspective of self-determination theory, academic self-efficacy moderates students’ subjective interpretation of the teacher-caring environment. Students with high self-efficacy are more likely to perceive teachers’ support, inclusiveness, and academic guidance, thereby enhancing their satisfaction of competence, autonomy, and relatedness needs, which amplifies learning motivation, encourages academic challenge-seeking, and facilitates classroom interaction [56]. However, it remains unclear how the combinations of conscientiousness, supportiveness, inclusiveness, academic ability efficacy, and academic behavioral efficacy systematically influence academic achievement. Given its focus on asymmetric and equifinal causal relationships [26], a configurational perspective is well-suited to analyzing how teacher caring behavior and academic self-efficacy interact to shape academic achievement.

**Fig. 1:**
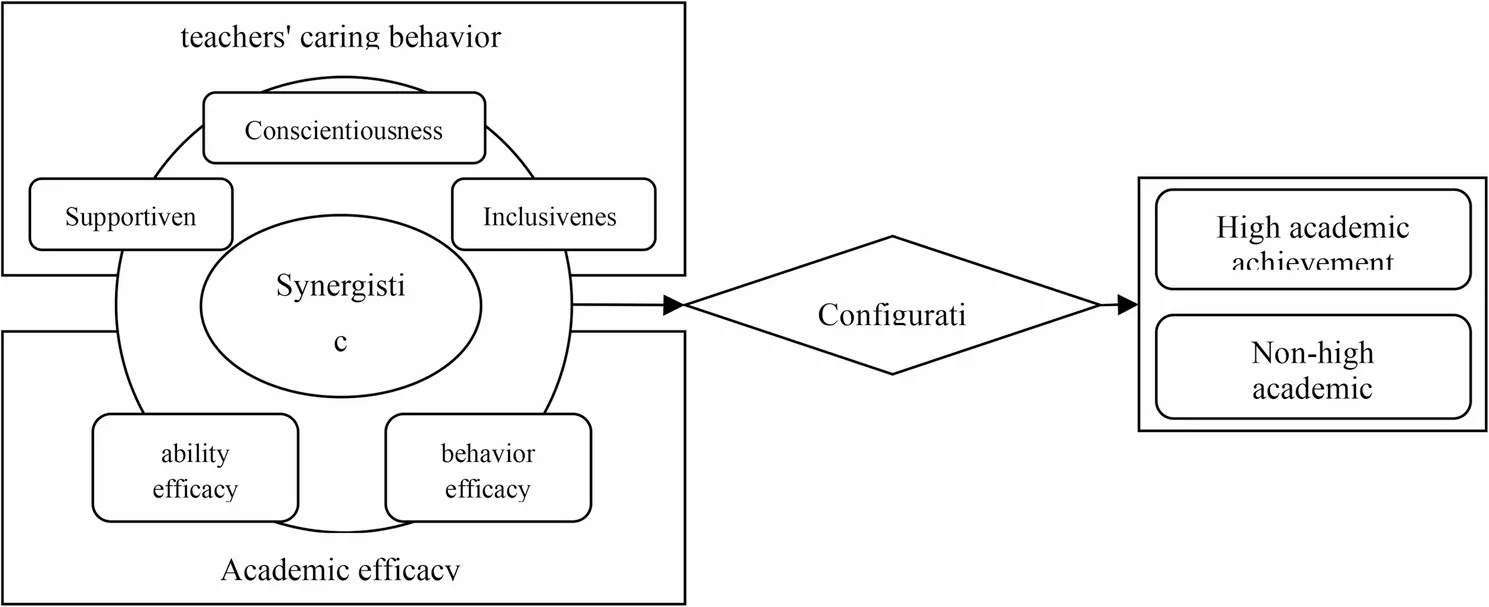
Theoretical model

In summary, how the five conditions—conscientiousness, supportiveness, inclusiveness, academic ability efficacy, and academic behavioral efficacy—interact in complex ways to influence academic achievement remains an open question. To address this, drawing upon social cognitive theory, this study integrates both external (teacher caring behavior) and internal (academic self-efficacy) factors to examine the multiple concurrent antecedents and causation complexity underlying academic achievement [26]. Specifically, we employ fsQCA to explore how the five antecedent conditions across two levels combine and match to produce academic outcomes, and we propose a theoretical model as depicted in Fig. 1. The external-level antecedents comprise conscientiousness, supportiveness, and inclusiveness, while the internal-level antecedents consist of academic ability efficacy and academic behavioral efficacy.

## Methodology

### Qualitative comparative analysis

Qualitative comparative analysis (QCA) is a set-theoretic method designed to examine the configurational effects of multiple factors on a given outcome [57]. The adoption of QCA in this study is motivated by three considerations. First, unlike conventional approaches such as structural equation modeling and regression analysis, QCA identifies multiple pathways leading to the same outcome, thereby enabling the analysis of multiple concurrent causalities and the interplay among variables in complex contexts [26, 58]. Second, the occurrence of high or non-high academic achievement does not stem solely from student-level factors, but rather from the interaction between student-level attributes and teacher caring behavior. Third, although multiple pathways may underlie high and non-high academic achievement, the specific configurations that produce desired outcomes remain unclear. Accordingly, this study takes conscientiousness, supportiveness, inclusiveness, academic ability efficacy, and academic behavioral efficacy as antecedent conditions and employs fsQCA to explore the causal complexity underlying high academic achievement.

### Data collection

To conduct empirical analysis, this cross-sectional survey was carried out from November 15 to December 10, 2024, at a key provincial agricultural university located in North China—Hebei Agricultural University, which features a complete discipline layout covering agriculture, engineering, management, humanities and social sciences, and possesses a student cohort with prominent rural-origin and practice-oriented learning characteristics distinctive from comprehensive or technical universities. We collected primary data via self-reported questionnaire surveys. All measurement variables were measured using mature, validated scales adopted from existing peer-reviewed literature.

To guarantee the rationality and reliability of questionnaire items, we drafted the initial measurement battery and subsequently invited multiple education management and psychology scholars to assess and revise the items. A pilot test with 48 undergraduate students was further implemented to refine ambiguous wording, confusing item descriptions and logical structures before formal data collection. A mixed sampling strategy integrating convenience sampling and snowball sampling was adopted to recruit participants. Specifically, an anonymous 20-minute digital questionnaire designed through Questionnaire Star (a mainstream professional online survey platform widely used in Chinese mainland academic research) was first distributed to college administrative and teaching staff on campus, who forwarded the survey link to eligible undergraduate students in their respective classes and research groups.

All collected responses were fully anonymized without any personally identifiable information (e.g., name, student ID, mobile number) to eliminate social desirability bias and protect participants’ privacy. Rigorous inclusion and exclusion criteria were set to enhance sample eligibility and representativeness: only full-time undergraduate students who voluntarily signed the informed consent form and agreed to participate were included; postgraduate and doctoral students were excluded to unify the research population and avoid heterogeneous learning experience interference. As the target university undertakes talent cultivation for rural revitalization in the Beijing-Tianjin-Hebei regional agrarian sector, its undergraduate sample can reflect the learning status, teacher-student interaction and academic development characteristics of agricultural university undergraduates in North China, improving the external validity of this study’s conclusions.

After eliminating incomplete, repetitive and carelessly filled questionnaires, 276 valid responses were retained for formal data analysis. Detailed demographic profiles of the valid sample are reported as follows: In terms of gender, male students made up 56.16% of participants, while female students constituted the remaining 43.84%. Regarding disciplinary background, students majoring in liberal arts and management disciplines accounted for 61.96%, and those majoring in science, engineering and agronomy took up 38.04%. For grade distribution, freshmen and sophomores represented 53.62% of the sample, and juniors and seniors accounted for 46.38%.

Moreover, to mitigate the threat of common method variance (CMV), we adopted three complementary diagnostic procedures as recommended by prior studies. First, we ran an unrotated exploratory factor analysis (EFA) following Podsakoff et al. [59]. The first extracted principal component with an eigenvalue greater than 1 explained merely 31.556% of the total variance, falling well below the 50% critical cutoff, which preliminarily ruled out severe CMV contamination [29]. Second, we implemented a confirmatory factor analysis (CFA) to test for CMV, following Podsakoff et al. [59]. The single-factor baseline model yielded unacceptable fit indices (χ²/df = 3.625, CFI = 0.702, GFI = 0.752, TLI = 0.664, RMSEA = 0.126). By contrast, the hypothesized six-factor measurement model achieved satisfactory model fit (χ²/df = 1.478, GFI = 0.904, CFI = 0.955, TLI = 0.939, RMSEA = 0.054), further evidencing negligible CMV bias. Third, we employed the unmeasured latent method factor technique proposed by Williams et al. [60]. A common latent method factor was incorporated into the original measurement model (Model T1) to construct a rival model (Model T2). Model comparisons revealed marginal fit differences between T1 (χ²/df = 1.478, GFI = 0.904, CFI = 0.955, RMSEA = 0.054) and T2 (χ²/df = 1.495, GFI = 0.923, CFI = 0.972, RMSEA = 0.048), with all changes in fit statistics (GFI, CFI, RMSEA) smaller than 0.02, signifying no meaningful fit improvement after accounting for method artifacts. Additionally, the average variance extracted (AVE) of the latent method factor only reached 17.58%, substantially lower than the 50% threshold. Collectively, these three tests confirmed that CMV exerted no substantial distorting effects on our empirical findings.

### Variable measurement

#### Teachers’ caring behavior

The caring behavior of teachers was gauged with the 14-item scale, borrowed from Lei et al. [13]. The proposed scale contains three major dimensions, namely (1): conscientiousness (6 items, such as, “I can seriously complete the classroom teaching”); (2) supportiveness (5 items, such as, “I can give detailed answers to students’ questions”); and (3) inclusiveness (3 items, including, “When a student’s viewpoint conflicts with mine, I respect their perspective”). Consistently, students were instructed to rate the items on a 5-point Likert scale that ranged from 1 (strongly disagree) to 5 (strongly agree). Besides, the overall Cronbach’s α of the full scale was 0.852, indicating satisfactory internal consistency. As illustrated in Table 1, the three subdimensions of teacher caring behavior—namely inclusiveness, supportiveness, and conscientiousness—also achieved acceptable Cronbach’s α values of 0.826, 0.743, and 0.726, respectively. In addition, the average variance extracted (AVE) values for conscientiousness, supportiveness, and inclusiveness were 0.537, 0.525, and 0.652, all exceeding the 0.5 critical threshold. Taken together, these findings confirm that the measurement scale possesses robust reliability and convergent validity.

**Table 1:** Convergent validity and reliability

Variables	Items	Validity			Reliability
		Factor loadings	CR	AVE	Cronbach’s α
Conscientiousness	C1	0.795	0.874	0.537	0.826
	C2	0.764			
	C3	0.759			
	C4	0.709			
	C5	0.690			
	C6	0.673			
Supportiveness	S1	0.764	0.847	0.525	0.743
	S2	0.741			
	S3	0.723			
	S4	0.703			
	S5	0.689			
Inclusiveness	I1	0.833	0.849	0.652	0.726
	I2	0.822			
	I3	0.765			
Academic ability efficacy	AAE1	0.781	0.835	0.558	0.736
	AAE2	0.753			
	AAE3	0.730			
	AAE4	0.723			
Academic behavior efficacy	ABE1	0.764	0.820	0.533	0.708
	ABE2	0.744			
	ABE3	0.725			
	ABE4	0.686			
Academic achievement	AA1	0.882	0.884	0.717	0.835
	AA2	0.872			
	AA3	0.846			

#### Academic self-efficacy

Academic self-efficacy was estimated with the help of an 8-items scale adapted from Huang & Wang [20]. Prominently, this scale contains two main dimensions: (1) ability self-efficacy (4 items, e.g., “I believe I can execute all the learning tasks assigned by the teacher”); and (2) behavior self-efficacy (4 items, such as., “When I think regarding a certain problem, I can apply the knowledge I have learned prior to and after thinking”). Thereafter, students were warranted to rate the items on a 5-point Likert scale that also ranged from 1 (strongly disagree) to 5 (strongly agree). Meanwhile, the overall Cronbach’s α of the scale was 0.804, which reflected satisfactory internal consistency. As presented in Table 1, the subscales of academic capability self-efficacy and academic behavior self-efficacy also attained acceptable Cronbach’s α values of 0.736 and 0.708, respectively. Furthermore, the AVE values for academic capability self-efficacy and academic behavior self-efficacy were 0.558 and 0.533, both surpassing the 0.5 critical cutoff. Collectively, these outcomes verify that the measurement scale possesses sound reliability and convergent validity.

#### Academic achievement

Explicitly, academic achievement was weighed with the help of 3 items adapted from Zhang et al. [61]. Accordingly, college students were requested to rate the items on a five-point Likert scale that varied from 1 (strongly disagree) to 5 (strongly agree). For instance, one sample item was “I will complete the learning task within the stipulated time period”, “My learning outcomes covering knowledge, competencies and literacy have achieved the expected goals.” ,“I maintain a high level of learning quality.” Additionally, as shown in Table 1, the Cronbach’s α coefficient of the overall scale reached 0.835, and the AVE value was 0.717, exceeding the critical threshold of 0.5. These results collectively demonstrate satisfactory reliability and convergent validity of the measurement scale.

### Correlation analysis

Table 2 presents descriptive statistics and correlation results, including the means, standard deviations (SDs), and pairwise correlations of all research variables. Consistent with theoretical expectations, academic achievement was significantly and positively correlated with conscientiousness (γ = 0.541, *p* < 0.05), supportiveness (γ = 0.471, *p* < 0.05), academic ability self-efficacy (γ = 0.558, *p* < 0.05), inclusiveness (γ = 0.308, *p* < 0.05), and academic behavior self-efficacy (γ = 0.504, *p* < 0.05). Furthermore, the 95% confidence intervals of all correlation coefficients excluded zero, and all correlation magnitudes were lower than 0.6. Multivariate regression analysis was subsequently conducted; the variance inflation factor (VIF) values ranged from 1.605 to 2.009, all below the cutoff of 3, confirming the absence of severe multicollinearity across variables [62]. It should be noted that the correlation analysis serves a primarily diagnostic and descriptive purpose rather than a hypothesis-testing one. Specifically, it provides an initial understanding of the bivariate associations among conditions and, more importantly, screens for excessively high correlations (i.e., > 0.7) that would indicate empirical redundancy among conditions—a prerequisite for valid configurational analysis. These diagnostic results, together with the VIF checks, confirm that the selected conditions are sufficiently distinct for inclusion in the subsequent fsQCA. While correlation analysis examines net linear relationships at the variable level, fsQCA complements this by exploring combinatorial and equifinal causal configurations among conditions, thereby offering a more nuanced understanding of the complex pathways leading to high academic achievement.

**Table 2:** Results of correlation analysis

Variable	Mean	SD	1	2	3	4	5	6	95% CI	
									Lower limit	Upper limit
1. Conscientiousness	4.061	0.555	1						0.465	0.653
2. Supportiveness	4.153	0.541	0.591^**^	1					0.457	0.625
3. Inclusiveness	3.583	1.069	0.223^**^	0.327^**^	1				0.476	0.572
4. Academic ability self-efficacy	4.177	0.555	0.588^**^	0.555^**^	0.262^**^	1			0.533	0.652
5. Academic behavior self-efficacy	4.073	0.594	0.485^**^	0.430^**^	0.234^**^	0.545^**^	1		0.504	0.605
6. Academic achievement	4.318	0.527	0.541^**^	0.471^**^	0.308^**^	0.558^**^	0.504^**^	1	0.996	1.142

*SD* standard deviation

**p < 0.01, *p < 0.05

## Results

### Calibration

Prior to conducting necessary condition analysis and sufficiency analysis, data calibration is required [63]. Calibration refers to the process of assigning set membership scores to each case [26], and it constitutes one of the core procedures for fsQCA. Data calibration in fsQCA analysis principally includes two main methods. On the one hand, the direct calibration method calibrates each variable through three anchor points determined directly; while, on the other hand, the second technique assigns values to data in combination with the actual distribution of samples, practical experience, or theoretical knowledge, which is referred to as the indirect calibration method [64]. Reportedly, all variables in this paper were estimated using a 5-point Likert scale, but since the samples were majorly derived in the form of self-reports, there may be certain subjective intentions. Therefore, 1, 3.5, and 5 cannot be mechanically chosen as the three anchor points that present the full non-membership threshold (0.05), crossover point (0.5), and null membership threshold (0.95) [64]. As a result, drawing on Jacobs and Cambré [65], the indirect calibration approach was implemented, and the “percentile” function in SPSS was employed to compute the values of 95%, 50%, and 5% [57]. Additionally, to avoid potential theoretical difficulties in the subsequent analysis, we followed Fiss’s [28] recommendation by adding 0.001 to calibrated membership scores of exactly 0.5, adjusting them to 0.501. The specific calibration anchors for both conditions and the outcome are presented in Table 3.

**Table 3:** Specific values of calibration anchor points of each variable

		Full non-membership threshold	Cross over point	Full membership threshold
Antecedent variable	Conscientiousness	3.000	4.167	4.833
	Supportiveness	3.200	4.200	4.800
	Inclusiveness	1.667	4.000	5.000
	Academic ability self-efficacy	3.000	4.250	5.000
	Academic behavior self-efficacy	3.000	4.250	5.000
Result variable	Academic achievement	3.250	4.500	5.000

### Necessary analysis

Prior to conducting the configurational analysis, a necessity analysis was performed to determine whether any single condition constitutes a necessary condition for the outcome. A condition is deemed necessary if the outcome occurs only when the condition is present and does not occur in its absence [58]. Consistency serves as the key indicator for assessing necessity; generally, a condition with a consistency level of 0.9 or above is considered necessary [58]. The necessity test results for each antecedent condition are presented in Table 4. As shown, no single condition exceeded the 0.9 consistency threshold [57], with consistency values ranging from 0.511 to 0.802, indicating that none constitutes a necessary condition for the occurrence of either high or non-high academic achievement [65]. Consistent with the configurational logic of fsQCA, this finding suggests that high academic achievement is not driven by any isolated factor, but rather emerges from the combined effect of multiple conditions—a pattern that will be further explored in the subsequent sufficiency analysis. Accordingly, we do not engage in detailed case-level discussion within the necessity analysis, as the absence of necessary conditions does not provide a meaningful basis for fine-grained case scrutiny.

**Table 4:** Necessity analysis results of antecedent conditions on outcome variables

Antecedent variable		High academic achievement		Non-high academic achievement	
		Consistency	Coverage	Consistency	Coverage
Teachers’ caring behavior	Conscientiousness	0.797	0.777	0.576	0.590
	~ Conscientiousness	0.579	0.566	0.782	0.801
	Supportiveness	0.817	0.729	0.603	0.564
	~ Supportiveness	0.511	0.551	0.710	0.802
	Inclusiveness	0.580	0.539	0.752	0.732
	~ Inclusiveness	0.711	0.733	0.526	0.568
Academic self-efficacy	Academic ability self-efficacy	0.802	0.746	0.598	0.583
	~ Academic ability self-efficacy	0.551	0.567	0.739	0.796
	Academic behavior self-efficacy	0.736	0.769	0.543	0.596
	~ Academic behavior self-efficacy	0.613	0.562	0.790	0.759

### Sufficient analysis of the conditional configuration

Following the recommendations of Ragin [64] and Du et al. [26], the consistency threshold was set at 0.800, the frequency cutoff at 3, and the PRI consistency threshold at 0.750 [26, 63]. The data were analyzed using fsQCA 3.0 software, yielding three types of solutions: complex, intermediate, and parsimonious. Core conditions were identified as those appearing in both parsimonious and intermediate solutions, while peripheral conditions were those appearing only in intermediate solutions [26]. The detailed results are presented in Table 5. Three configurational pathways (A1, A2, and A3) were identified for high academic achievement, with individual consistencies of 0.866, 0.903, and 0.892, respectively. The overall solution consistency reached 0.846, exceeding the recommended threshold of 0.75 and thus meeting established methodological standards [26, 64]. These results indicate that, for most cases, the three configurations represent sufficient conditions for achieving high academic achievement. The overall coverage was 0.620, accounting for 62% of high academic achievement. For non-high academic achievement, two configurational pathways (B1, B2) were identified, each with a consistency of 0.921 and an overall consistency of 0.910, suggesting that these two configurations represent sufficient conditions for low academic achievement. The overall coverage was 0.593, explaining 59.3% of the underlying reasons for low academic achievement. Collectively, the consistency values for all configurations of high and low academic achievement exceeded the 0.800 threshold, demonstrating that fsQCA effectively identified five configurations for both high and non-high academic achievement with strong explanatory power.

**Table 5:** Configurations for high and non-high academic achievement in fsQCA

Antecedent variable	High academic achievement			Non-high academic achievement	
	A1	A2	A3	B1	B2
Conscientiousness		•	⚫	⊗	⊗
Supportiveness	⚫	⚫		⊗	
Inclusiveness	⊗	⊗	⊗		⚫
Academic ability self-efficacy	⚫		⚫	⊗	⊗
Academic behavior self-efficacy		⚫	•	⊗	⊗
Consistency	0.866	0.892	0.903	0.921	0.921
Raw coverage	0.574	0.511	0.520	0.533	0.542
Unique coverage	0.081	0.019	0.027	0.051	0.060
Solution consistency	0.846			0.910	
Solution coverage	0.620			0.593	

⚫ indicate the core conditions, • indicate the peripheral conditions, ⊗ indicate its absence, Blank spaces indicate that the dimension has no effect on the result

#### Configuration analysis for high academic achievement

As shown in Table 4, three configurational pathways (A1, A2, and A3) were identified for high academic achievement, each labeled following the principles of parsimony, holistic capture, and essence evocation [63]. Guided by configurational theorizing and the analysis of antecedent conditions, each pathway was interpreted and named accordingly. In Configuration A1, supportiveness and academic ability efficacy jointly serve as driving forces, with conscientiousness and academic behavioral efficacy as peripheral conditions, and inclusiveness absent. This configuration accounts for 57.4% of the high-achievement subsample and is thus labeled the “Supportiveness–Ability Self-Efficacy Driven Model.” Configuration A2 features supportiveness and academic behavioral efficacy as core conditions, with conscientiousness as peripheral. Covering 51.1% of the high-achievement subsample, it is termed the “Conscientiousness–Supportiveness–Behavioral Self-Efficacy Driven Model.” Configuration A3 takes conscientiousness and academic ability efficacy as core conditions, with academic behavioral efficacy as peripheral and inclusiveness absent. This pathway explains 52% of the high-achievement subsample and is designated the “Responsibility–Self-Efficacy Driven Model.”

#### Configuration analysis for non-high academic achievement

Solutions B1 and B2 present the configurations for low academic achievement (see Table 5), where the presence or absence of each condition depends on their combinatorial patterns. Configuration B1 indicates that regardless of perceived teacher inclusiveness, students who fail to perceive high conscientiousness, high supportiveness, high academic ability efficacy, and high academic behavioral efficacy tend to report low academic achievement. This solution accounts for 55.3% of the cases in the low-achievement subsample. Configuration B2, in turn, identifies inclusiveness as a crucial determinant of low academic achievement, particularly when students do not perceive high conscientiousness or report low levels of academic ability efficacy and academic behavioral efficacy. The raw coverage of solution B2 is 54.2%.

### Robustness test

Following the recommendations of Zhang and Du [58], we conducted robustness tests by adjusting the consistency threshold, PRI consistency threshold, and case frequency threshold. First, we raised the consistency level from 0.8 to 0.85, and the resulting configurations for academic achievement were identical to those obtained prior to the adjustment (see Table 6). Second, we increased the PRI consistency threshold from 0.75 to 0.78, and the results remained consistent with the original findings (see Table 7). Third, we raised the case frequency threshold from 3 to 4; the resulting configurations (A1’, A2’, A3’) were largely consistent with the original configurations (A1, A2, A3), and the configurations for non-high academic achievement, along with their overall solution consistency and coverage, remained unchanged (see Table 8). Taken together, these results confirm the robustness of our findings.

**Table 6:** Robustness test with an elevated consistency threshold

Antecedent variable	High academic achievement			Non-high academic achievement	
	A1	A2	A3	B1	B2
Conscientiousness		•	⚫	⊗	⊗
Supportiveness	⚫	⚫		⊗	
Inclusiveness	⊗	⊗	⊗		⚫
Academic ability self-efficacy	⚫		⚫	⊗	⊗
Academic behavior self-efficacy		⚫	•	⊗	⊗
Consistency	0.866	0.892	0.903	0.921	0.921
Raw coverage	0.574	0.511	0.520	0.533	0.542
Unique coverage	0.081	0.019	0.027	0.051	0.060
Solution consistency	0.846			0.910	
Solution coverage	0.620			0.593	

⚫ indicate the core conditions, • indicate the peripheral conditions, ⊗ indicate its absence, Blank spaces indicate that the dimension has no effect on the result

**Table 7:** Robustness test with an elevated PRI value

Antecedent variable	High academic achievement			Non-high academic achievement	
	A1	A2	A3	B1	B2
Conscientiousness		•	⚫	⊗	⊗
Supportiveness	⚫	⚫		⊗	
Inclusiveness	⊗	⊗	⊗		⚫
Academic ability self-efficacy	⚫		⚫	⊗	⊗
Academic behavior self-efficacy		⚫	•	⊗	⊗
Consistency	0.866	0.892	0.903	0.921	0.921
Raw coverage	0.574	0.511	0.520	0.533	0.542
Unique coverage	0.081	0.019	0.027	0.051	0.060
Solution consistency	0.846			0.910	
Solution coverage	0.620			0.593	

⚫ indicate the core conditions, • indicate the peripheral conditions, ⊗ indicate its absence, Blank spaces indicate that the dimension has no effect on the result

**Table 8:** Robustness test with an elevated case frequency threshold

Antecedent variable	High academic achievement			Non-high academic achievement	
	A1’	A2’	A3’	B1’	B2’
Conscientiousness		⚫	⚫	⊗	⊗
Supportiveness	⚫	⚫		⊗	
Inclusiveness	⊗	⊗	⊗		⚫
Academic ability self-efficacy	⚫		⚫	⊗	⊗
Academic behavior self-efficacy		•	⚫	⊗	⊗
Consistency	0.866	0.884	0.913	0.921	0.921
Raw coverage	0.574	0.521	0.514	0.533	0.542
Unique coverage	0.081	0.019	0.027	0.051	0.060
Solution consistency	0.846			0.910	
Solution coverage	0.620			0.593	

⚫ indicate the core conditions, • indicate the peripheral conditions, ⊗ indicate its absence, Blank spaces indicate that the dimension has no effect on the result

## Discussion

### Theoretical implications

First, drawing upon social cognitive theory, this study constructs a two-level theoretical framework incorporating teacher caring behavior and academic self-efficacy to investigate the mechanisms underlying academic achievement, thereby deepening the understanding of key antecedents and offering a systematic configurational analytical framework for the formation of academic achievement. Although academic achievement has long attracted scholarly attention and research on its enhancement mechanisms continues to accumulate [66–68], prior studies have predominantly focused on a single level—either internal factors or external contexts—with limited attention to the synergistic interplay between teacher caring behavior and academic self-efficacy. Research on cross-level enhancement mechanisms remains scarce, and the pathways through which multi-level factors jointly shape academic achievement are not well understood [69]. Neglecting the cross-level nature of these determinants hinders a comprehensive grasp of the driving mechanisms of academic achievement. Given that existing studies have recognized the multi-level nature of its antecedents [33], this study adopts a configurational perspective to establish a cross-level model that integrates internal and external factors, further exploring the preconditions for academic achievement. In doing so, it not only addresses the limitations of prior fragmented investigations into the formation mechanisms of academic achievement but also elucidates the synergistic roles of teacher caring behavior and academic self-efficacy in enhancing academic outcomes, responding to recent calls for multi-level research in educational psychology [32]. These findings contribute to a more systematic analytical framework for understanding the mechanisms that drive academic achievement.

Second, by employing fsQCA, this study uncovers multiple pathways to academic achievement, extending the application of this method to the field of academic achievement research and systematically analyzing the complex relationships among teacher caring behavior, academic self-efficacy, and academic outcomes. On the one hand, the use of fsQCA allows for an in-depth exploration of the synergistic mechanisms underlying high academic achievement, thereby enriching theoretical perspectives on academic achievement while responding to calls for applying fsQCA to the study of complex phenomena [63]. On the other hand, although extant research has extensively examined academic achievement through quantitative approaches [13, 70], conventional regression methods predominantly focus on the net effects of individual predictors. Departing from this tradition, the present study adopts a configurational perspective that fully accounts for the complexity of configurational causation [71], integrating both teacher caring behavior and academic self-efficacy to investigate how combinatorial conditions shape academic achievement. Three distinct configurations for high academic achievement were identified: the “Conscientiousness–Supportiveness–Behavioral Self-Efficacy Driven Model,” the “Conscientiousness–Self-Efficacy Driven Model,” and the “Supportiveness–Ability Self-Efficacy Driven Model.” These findings not only mitigate the inconsistencies in prior research that often overlooked multi-level determinants [72], but also confirm that academic achievement does not hinge on any single factor in isolation—corroborating Sheng et al.‘s assertion that the antecedents of academic achievement function configurally, such that a given factor may exert promoting or inhibiting effects only when combined with others [32]. Furthermore, this study demonstrates the advantages of fsQCA in explaining the interplay among multiple factors, transcending the limitations of conventional analytical approaches [28] and offering novel methodological insights for future research on the mechanisms and pathways underlying academic achievement enhancement.

Third, the necessity analysis reveals that no single condition—conscientiousness, supportiveness, inclusiveness, academic ability efficacy, or academic behavioral efficacy—constitutes a necessary condition for high academic achievement; rather, the antecedents of academic achievement operate configurally. This finding diverges from prior studies employing conventional regression approaches. For instance, Lei et al. [13], Yang et al. [14], and Shao et al. [17] reported that teacher caring behavior directly predicts academic performance, which is inconsistent with the three configurational pathways identified in this study. Conversely, Zhou and Kang [23] found that the direct effect of teacher support on English academic performance was statistically non-significant; however, our results suggest that when internal factors are considered, teacher support effectively enhances academic achievement. Specifically, configurations A1 and A2 indicate that high academic achievement emerges under conditions of both high teacher support and high academic self-efficacy. According to the general learning model [73], teacher support functions as a contextual variable that influences academic outcomes only through the mediating role of individual efficacy beliefs. Furthermore, although some studies have found inclusiveness to promote academic achievement [13], our findings demonstrate that high academic achievement can also occur under low inclusiveness. In configuration A3, when both supportiveness and academic ability efficacy are high, academic achievement is enhanced even in the presence of low inclusiveness. This suggests that when teacher inclusiveness is low, students’ strong confidence, internal locus of control, and achievement orientation—combined with teacher conscientiousness and self-efficacy—may drive them to creatively complete learning tasks in accordance with teacher expectations, thereby yielding superior academic outcomes. In addition, while some research has found that self-efficacy does not significantly predict academic achievement in general learning or vocational learning contexts [24], our results indicate that high academic self-efficacy can also lead to high achievement. In configuration A1, when college students exhibit high academic ability efficacy, in concert with teacher supportiveness, high academic achievement is attainable. Consistent with social cognitive theory, high academic achievement is likely to occur when students possess both strong academic self-efficacy and perceived teacher support. Collectively, these configurational findings provide a more fine-grained explanation of the pathways to high academic achievement, shedding light on the differential emergence of high achievement and the interdependent configurational effects among conditions.

### Managerial implications

First, the approach to enhancing college students’ academic achievement should shift from a “targeted optimization” mindset to a “configurational coordination” perspective. Drawing on the fsQCA configurational framework [58, 63], this study integrates teacher caring behavior and self-efficacy into a unified model, promoting deep synergy between “teaching” and “learning,” and yielding three differentiated pathways to success. Universities should avoid one-size-fits-all teaching management practices and instead allocate limited educational resources to the most appropriate driving models. Emphasis should be placed on “how teaching can activate and align with student learning,” thereby fostering a virtuous cycle of mutual enhancement between instruction and learning. This approach provides a systematic, “combination” intervention strategy for the differentiated formation and improvement of college students’ academic achievement.

Second, practical application strategies for higher education institutions based on the three configurations. In teaching practice, universities should flexibly apply the following intervention strategies according to the underlying logic of each configuration, targeting different student groups, course types, and learning stages.

#### The “Conscientiousness–Supportiveness–Behavioral Self-Efficacy Driven Model”

This configuration requires teachers to be both highly conscientious and highly supportive, while students exhibit high behavioral self-efficacy. It is particularly applicable to freshman transition periods, foundational core courses, or long-term learning tasks requiring sustained effort (e.g., thesis writing, professional internships). From the perspective of teacher behavior intervention, universities should encourage teachers to adopt a dual role—“strict mentor and caring guardian”—in such contexts. Teachers should establish clear teaching standards and assessment milestones (conscientiousness), while also providing timely guidance, emotional support, and resource allocation when students encounter difficulties (supportiveness). From the perspective of student efficacy, universities should prioritize behavioral efficacy training, such as offering time management workshops, implementing goal-setting and check-in mechanisms, and conducting resilience education, to enhance students’ executive capacity and persistence, thereby aligning their behavioral self-efficacy with teachers’ dual care.

#### The “Conscientiousness–Self-Efficacy Driven Model”

This configuration requires teachers to be highly conscientious, with students possessing high self-efficacy (whether ability- or behavior-oriented). Notably, supportiveness and inclusiveness are not core conditions in this configuration. This suggests that for students with strong intrinsic drive and high confidence, teachers’ rigorous and responsible approach alone suffices to drive high achievement; excessive emotional support or inclusiveness may instead lower academic standards or foster dependency. This model is well-suited for top-tier talent development programs (e.g., “Strengthening Basic Disciplines Plan,” honors programs), advanced seminar courses for senior students, and coaching for high-level disciplinary competitions. From the perspective of teacher behavior intervention, universities should guide teachers to implement “high standards and strict requirements” in academic mentorship. Teachers should focus their efforts on introducing cutting-edge knowledge, ensuring methodological rigor, and training critical thinking (conscientiousness), while minimizing unnecessary “over-care” or “standard-lowering inclusiveness.” From a management perspective, in allocating mentors for exceptional students, universities should prioritize pairing them with academically rigorous and stringent supervisors, thereby maximizing the academic potential of highly self-efficacious students through the “strict mentor produces outstanding students” configurational effect.

#### The “Supportiveness–Ability Self-Efficacy Driven Model”

This configuration entails high teacher supportiveness coupled with high academic ability self-efficacy among students. It suggests that when students possess strong confidence in their own abilities, teachers’ provision of extensive platforms and resources can be rapidly translated into high academic achievement. This model is particularly applicable to innovation and entrepreneurship education, interdisciplinary project-based learning, research training programs (e.g., undergraduate innovation projects), and interventions for “gifted but under-motivated” students who demonstrate potential yet lack immediate drive. From the perspective of teacher behavior intervention, universities should encourage teachers to adopt the role of “resource providers” and “platform builders.” Rather than micromanaging details, teachers should offer rich access to literature, laboratory equipment, off-campus practical opportunities, and the freedom for exploration. From the perspective of student efficacy intervention, universities should strengthen students’ confidence in their own capabilities through formative assessment and diversified presentation platforms. For instance, academic salons and innovation achievement exhibitions can be organized to provide students with positive feedback, thereby reinforcing their identification with their own learning abilities.

Third, there is also due need to emphasize the significance of non-high academic achievement configuration. Primarily, the configuration path of high and non-high academic achievement is asymmetrical, and the opposite of the cause of non-high academic achievement is the cause of high academic achievement. Moreover, in order to improve academic achievement, it is essential to utilize configuration cognition to deeply comprehend the complex association between teachers’ caring behavior and college students’ sense of self-efficacy and to discover and grasp the core elements from multiple paths [58]. Meanwhile, this paper reports that the lack of academic self-efficacy and conscientiousness presents a critical reason behind the lack of academic achievement. Resultantly, on the one hand, it is both urgent and crucial for universities and colleges to strengthen the conscientious behavior of teachers. Further, there is not only a need to motivate teachers to concrete their sense of responsibility in education and teaching at the policy level but also to enhance their sense of dedication through training and learning [74]. On the other hand, it is also highly imperative to nurture the academic self-efficacy of college students [75]. Consistently, teachers should extend more supportiveness and affiliations with college students, in order to encourage students to enhance their academic self-efficacy [76]. Besides, it is also imperative to guide college students to make correct attributions and help them analyze and address potential problems; support and guide college students to actively participate in learning activities, feel learning practices, carry out self-evaluations and others’ evaluations, and uplift positive experiences.

## Conclusion and limitations

### Conclusions

This study examined the configurational causation effects of conscientiousness, supportiveness, inclusiveness, academic ability self-efficacy, and academic behavioral self-efficacy on college students’ academic achievement. To the best of our knowledge, this is one of the earliest attempts to investigate the complex mechanisms underlying college students’ academic achievement using fsQCA. Although no single antecedent condition was found to be sufficiently necessary to explain either high or low academic achievement, it is noteworthy that—unlike inclusiveness—conscientiousness, supportiveness, academic ability self-efficacy, and academic behavioral self-efficacy emerged as sufficient conditions for high academic achievement. Conversely, the absence of inclusiveness appears to be a significant factor associated with low academic achievement. These findings underscore the critical roles of conscientiousness, supportiveness, academic ability self-efficacy, and academic behavioral self-efficacy in shaping college students’ academic outcomes. To effectively enhance academic achievement among college students, a holistic approach that simultaneously addresses all the aforementioned dimensions is warranted.

### Limitations

This study has several limitations. First, academic achievement is influenced by a multitude of factors; this study, grounded in social cognitive theory, incorporated only teacher caring behavior and academic self-efficacy into the theoretical model, potentially omitting variables such as major, socioeconomic status, and prior achievement. Future research should consider multi-level factors to more comprehensively explore the pathways to academic achievement. Second, regarding sample collection, this study employed convenience sampling via the Questionnaire Star platform, which may introduce sample bias and limit the generalizability of the findings. Future studies should adopt a combination of online and offline data collection methods based on random sampling principles to enhance sample quality and representativeness. Third, this study employed fsQCA to identify the interplay among multiple factors affecting academic achievement. Given that different types of universities may vary, and that students’ emphasis on academic achievement may differ accordingly, future research could distinguish between institution types—such as research universities and vocational colleges—and conduct comparative studies to reveal differential pathways to academic achievement across diverse higher education settings.

## Data Availability

The datasets used and/or analysed during the current study are available from the corresponding author on reasonable request.
